# Materia Medica and Therapeutics

**Published:** 1877-08

**Authors:** 


*A Practical Treatise on Materia Medica and Therapeutics.* By ROBERTS BARTHOLOW, A. M. M. D., Professor of the Theory and Practice of Medicine and of Clinical Medicine and formerly Professor of Materia Medica and Therapeutics in the Medical College of Ohio, &c., &c., &c. New York: D. Appleton & Company, 549 and 551 Broadway, 1877. pp. 516.

This volume is fresh and pleasing in arrangement. original in its scheme of classification, and, as a whole comprehensive and desirable. The newer remedies are appropriately grouped with the old and for some of the old, new uses or restrictions are suggested. It does not aim to rival Wood or Stillé in exhaustiveness, but contains that information which is most essential to a skilful use of the armament of followers of Æsculapius. The chapter on alcohol and wines and the presentation of the different so-called cures, as grape cure, milk cure, &c., together with the location of all the important mineral springs of the world, and composition of their waters, are features of interest. The French and German as well as English names of each drug are given and as a heading to whatever follows with regard to it, a semi tabulated statement of its different preparations and the appropriate dose. This is a convenient arrangement for quick reference. Having become familiar with the contents of the book one would scarcely be willing to miss it from the library. We are glad the author was sufficiently undaunted with the fine things already done by American writers in this department, to give us this comparatively short, but useful treatise. M. B. M.

